# Prostate imaging scoring systems: indications, interpretation, and clinical impact

**DOI:** 10.1093/bjro/tzag021

**Published:** 2026-10-16

**Authors:** Emanuele Messina, Antonella Borrelli, Georgios Agrotis, Giorgio Brembilla, Maarten de Rooij, Ludovica Laschena, Francesca Mezzapesa, Ailin Dehghanpour, Francesco Giganti, Ivo G Schoots, Valeria Panebianco

**Affiliations:** Department of Radiological Sciences, Oncology and Pathology, Sapienza University/Policlinico Umberto I, Rome, 00161, Italy; Department of Radiological Sciences, Oncology and Pathology, Sapienza University/Policlinico Umberto I, Rome, 00161, Italy; Department of Radiology, The Netherlands Cancer Institute, Amsterdam, 1066, The Netherlands; GROW School for Oncology and Reproduction, Maastricht University, Maastricht, 6229, The Netherlands; Department of Radiology, IRCCS San Raffaele Scientific Institute, Vita-Salute San Raffaele University, Milan, 20132, Italy; Department of Medical Imaging, Radboud University Medical Center, Nijmegen, 6525, The Netherlands; Department of Radiological Sciences, Oncology and Pathology, Sapienza University/Policlinico Umberto I, Rome, 00161, Italy; Department of Radiological Sciences, Oncology and Pathology, Sapienza University/Policlinico Umberto I, Rome, 00161, Italy; Department of Radiological Sciences, Oncology and Pathology, Sapienza University/Policlinico Umberto I, Rome, 00161, Italy; Department of Experimental Medicine, Sapienza University/Policlinico Umberto I, Rome, 00161, Italy; Department of Radiology, University College London Hospitals, NHS Foundation Trust, London, NW1 2BU, United Kingdom; Division of Surgery & Interventional Science, University College London, London, WC1E 6BT, United Kingdom; Department of Radiology, The Netherlands Cancer Institute, Amsterdam, 1066, The Netherlands; Department of Radiology & Nuclear Medicine, Erasmus University Medical Center, Rotterdam, 3015, The Netherlands; Department of Radiological Sciences, Oncology and Pathology, Sapienza University/Policlinico Umberto I, Rome, 00161, Italy

**Keywords:** Prostate Cancer, Imaging, MRI, Scoring Systems

## Abstract

Prostate imaging, and especially multiparametric magnetic resonance imaging (mpMRI), has become central to the diagnosis, risk stratification, treatment planning, and follow-up of prostate cancer. Its widespread adoption has driven the development of structured imaging scoring systems designed to standardize acquisition, interpretation, and reporting across diverse clinical scenarios. This narrative review summarizes the principles, clinical applications, strengths, and limitations of the main prostate imaging scoring systems currently in use. These include PI-RADS for cancer detection, PRECISE for monitoring patients on active surveillance, PI-RR for post-treatment local recurrence, PI-FAB and TARGET for assessment after focal therapy, Node-RADS for lymph node evaluation, MET-RADS-P for whole-body MRI assessment of metastatic disease, PSMA-RADS for molecular imaging interpretation, and PI-QUAL for prostate MRI quality assurance. Collectively, these systems provide a shared diagnostic language that improves inter-reader agreement, supports evidence-based decision-making, and facilitates multidisciplinary care. Despite their proven clinical impact, challenges remain, including inter-reader variability, heterogeneous MRI quality, limited access to advanced imaging, and incomplete validation of emerging tools. Future directions include the incorporation of artificial intelligence, hybrid PET/MRI, and combined clinical-genomic biomarkers, together with prospective multicenter validation, to advance precision, reproducibility, and personalization in prostate cancer imaging.

## Introduction

Prostate cancer remains one of the most significant oncologic challenges worldwide, both in terms of incidence and clinical complexity.^1^ Over the past decade, multiparametric MRI (mpMRI) has radically transformed how prostate cancer is detected, characterized, and monitored. What began as a promising adjunct to conventional diagnostics has evolved into a core element of modern prostate cancer care, influencing biopsy decisions, therapeutic planning, and treatment response assessment. The integration of MRI into routine clinical pathways has been supported by robust evidence from randomized clinical trials demonstrating its superior ability to identify clinically significant prostate cancer (csPCa) while reducing unnecessary biopsies^2^ and limiting the detection of indolent disease.^3–8^ As a result, major international guidelines now endorse MRI as the preferred triage test in biopsy-naïve men, defining the so-called “MRI Pathway”.^3^^,^^9–12^

However, the rapid adoption of MRI in the diagnostic work-up of prostate cancer introduced a growing need for structured and reproducible interpretation. Variability in acquisition parameters, reader expertise, reporting terminology, and specific thresholds for the likelihood of csPCa historically limited MRI’s reliability across centers. To address these disparities, several scoring systems have been developed, each of which targets a distinct clinical scenario, the first represented by the Prostate Imaging Reporting and Data System (PI-RADS) score, which provides a unified algorithm for lesion detection and characterization, setting recommendations for image acquisition and scanner parameters.^13^ These scores reflect the expanding technological landscape and the growing role of hybrid imaging modalities.

These systems collectively anchor prostate imaging interpretation within precise decision-making algorithms, enabling harmonization across institutions and creating a common language for radiologists, urologists, oncologists, radiation oncologists, and multidisciplinary teams.

This review synthesizes the available evidence underlying each major prostate imaging scoring system, describes their clinical applications, and outlines challenges and future directions for further refinement. The goal is to provide a comprehensive overview of the prostate imaging scoring ecosystem, emphasizing its central role in delivering precise, reproducible, and patient-centered oncologic care.

## Diagnostic and monitoring phase

### PI-RADS: a core diagnostic framework

#### Structure and principles

The Prostate Imaging Reporting and Data System (PI-RADS) is the cornerstone of contemporary prostate MRI interpretation. PI-RADS was introduced to address major inconsistencies in acquisition standards and reporting practices. By defining standardized sequences, zonal anatomy considerations, and a uniform lexicon for lesion description, PI-RADS established a reproducible and clinically actionable diagnostic tool.^13–15^

PI-RADS v2.1, the current version, integrates information from T2-weighted imaging (T2WI), diffusion-weighted imaging (DWI) with corresponding apparent diffusion coefficient (ADC) maps, and dynamic contrast-enhanced imaging (DCE). The system assigns a score from 1 to 5 based on the likelihood of csPCa, with higher scores indicating a greater risk of significant disease. Crucially, PI-RADS is explicitly zonal: DWI is the dominant sequence in the peripheral zone, while T2WI guides assessment in the transition zone.^13^ DCE plays a secondary role, limited to upgrading equivocal lesions in the peripheral zone.^16^

The zonal weighting reflects the differing physiologic and histologic characteristics of each region. Peripheral tumors typically demonstrate diffusion restriction and subtle T2 signal loss, while transition-zone tumors must be discriminated from benign prostatic hyperplasia, which requires careful morphological analysis on T2WI^13^ (Figure 1).

**Figure 1:**
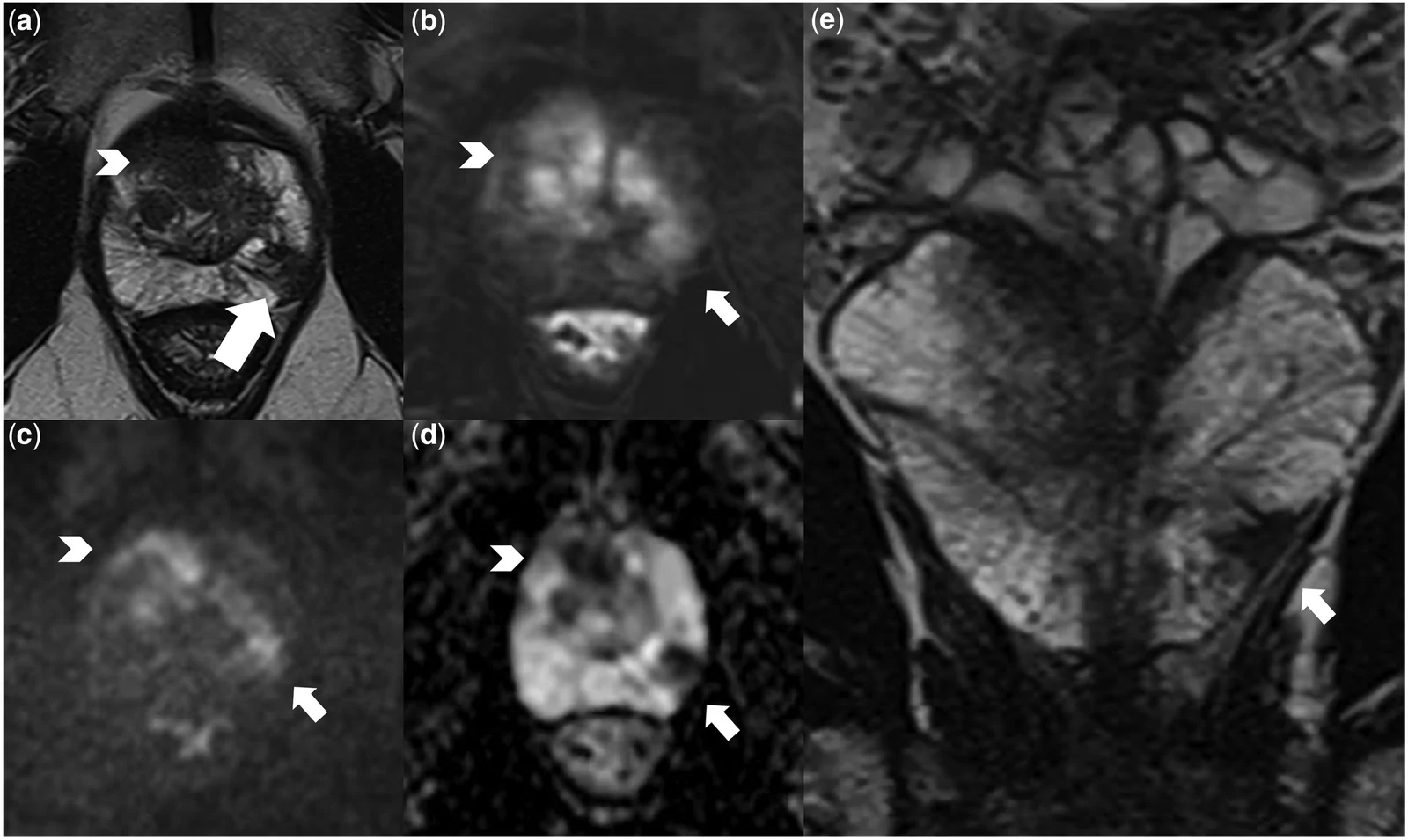
A 62-year-old man underwent multiparametric MRI following an episode of prostatitis, treated with antibiotics, and a rising PSA level (5.3 ng/mL). (a, e) Axial and coronal T2-weighted images show 2 lesions, located respectively in the right anterior apical transitional zone and in the left apical peripheral zone (white arrowhead and white arrow, respectively). (c, d) DWI (*b* = 2000) and the ADC map demonstrate marked diffusion restriction (white arrowhead). (b) DCE imaging shows early enhancement of both lesions. The first lesion was classified as PI-RADS 5 and the second as PI-RADS 4. Both lesions were biopsied using transrectal targeted FUSION biopsy, and the histopathology confirmed the presence of clinically significant prostate cancer, ISUP 3 (GS 4 + 3). Image quality was optimal, and the examination was graded as PI-QUAL 3/3 according to PI-QUAL v2. Abbreviations: ADC map = apparent diffusion coefficient map, DCE = dynamic contrast enhancement, DWI = diffusion-weighted imaging, MRI = magnetic resonance imaging, PI-QUAL = prostate imaging quality, PI-RADS = prostate imaging-reporting and data system.

#### Diagnostic accuracy and clinical impact

The clinical performance of PI-RADS is well established. Large meta-analyses report pooled sensitivity and specificity of approximately 87% and 74%, respectively, for detection of csPCa.^17^ Its integration into diagnostic pathways has been clinically transformative. The PROMIS trial demonstrated that mpMRI has superior sensitivity compared to transrectal ultrasound-guided biopsy, reducing unnecessary biopsies while maintaining safety.^11^ The PRECISION trial confirmed that an MRI-directed biopsy strategy improves the detection of clinically significant cancer while reducing detection of indolent tumors.^3^ On a meta-analysis, csPCa detection rates were as follows: PI-RADS 1, 6%; PI-RADS 2, 5%; PI-RADS 3, 19%; PI-RADS 4, 54%; and PI-RADS 5, 84%.^18^ The adoption of PI-RADS has also strengthened MRI-directed biopsy workflows. Targeted biopsy of PI-RADS 4-5 lesions yields high detection rates of csPCa, and systematic biopsy can be omitted in select patients, particularly those with negative MRI and low clinical risk.^4^

#### Limitations and areas of development

Despite its achievements, PI-RADS still faces several limitations. Indeed, the inter-reader agreement among radiologists of different level of experience is substantial to excellent, even if lower among general radiologists.^19^ Agreement for index-lesion identification reached an agreement coefficient (AC1) of 0.74 (95% CI, 0.70-0.78), with positive specific agreement of up to 93% for clinically significant disease among dedicated readers.^19^ PI-RADS 3 remains a diagnostic gray zone with limited clinical guidance; decisions frequently rely on PSA density, patient age, family history, or genomic assays.^16^ The role of DCE is debated, with MRI without contrast medium (biparametric MRI, bpMRI) being increasingly explored as a time-efficient and less-invasive alternative to the full MRI protocol.^20^ Recently, the PRIME Trial demonstrated that non-contrast bpMRI is noninferior to multiparametric MRI when considering the detection of csPCa.^21^

### PRECISE: monitoring patients during active surveillance

#### Structure and principles

Active surveillance (AS) has become the recommended strategy for men with low- or favorable intermediate- risk PCa, balancing oncologic safety with the preservation of quality of life and avoidance of overdiagnosis and overtreatment. The expanding role of MRI in AS is one of the most significant developments of modern prostate cancer care. MRI not only guides confirmatory biopsy but also provides a noninvasive method for monitoring disease over time. However, distinguishing true progression from benign fluctuation is not a trivial task.^22^

The PRECISE (Prostate Cancer Radiological Estimation of Change in Sequential Evaluation) scoring system was developed to provide a uniform structure for assessing longitudinal change, with the most recent iteration, PRECISE v2. It assigns a score from 1 to 5, reflecting the probability of disease regression (1-2), stability (3), which is stratified in visible (3-V) and non-visible (3-NonV), or progression (4-5) on serial imaging (Figure 2). In PRECISE v2 a new score (PRECISE X) was introduced to reflect the impossibility to provide PRECISE score when image quality is inadequate.

**Figure 2:**
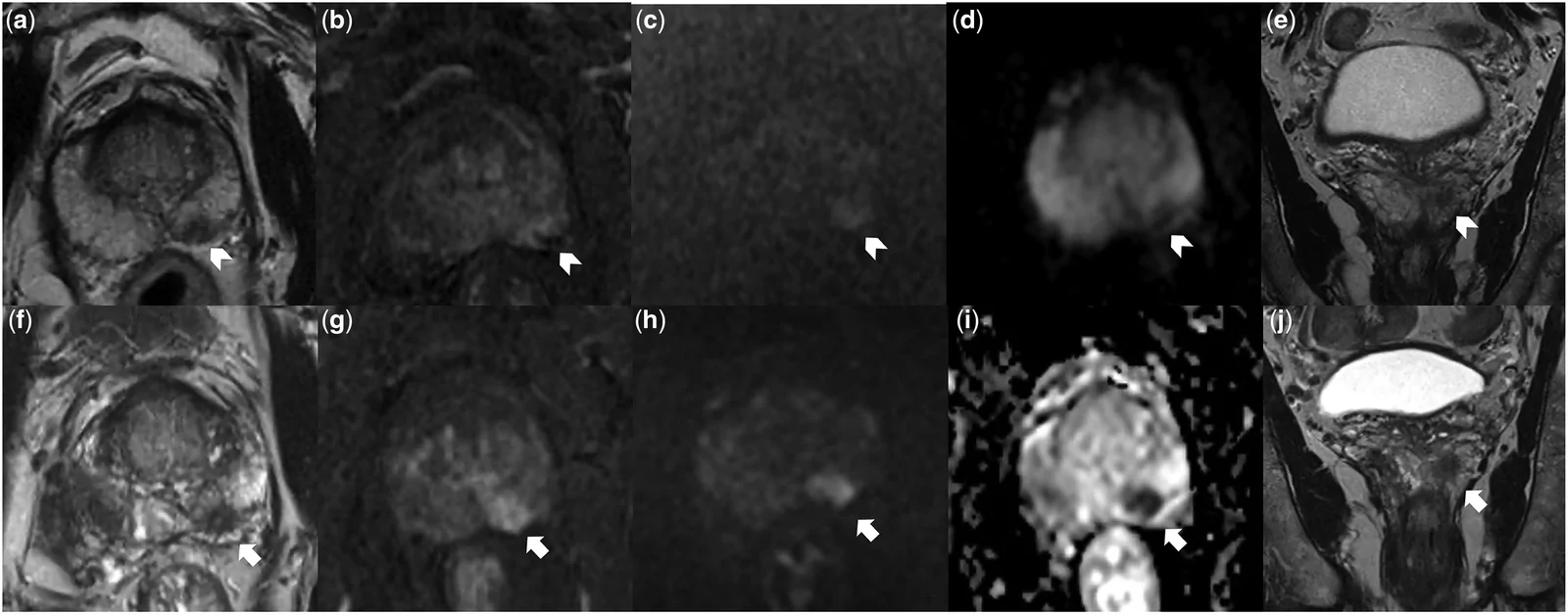
A 68-year-old man underwent multiparametric MRI, with a rising PSA level (7.4 ng/mL) (a, e) Axial and coronal T2WI show a hypointense lesion on the left mid-apical peripheral zone (white arrowhead). (c, d) DWI (*b* = 2000) and the ADC map demonstrate mild diffusion restriction (white arrowhead). (b) DCE imaging shows early enhancement (white arrowhead). The lesion was classified as PI-RADS 4 and underwent transrectal targeted FUSION biopsy, which revealed a Gleason score 6 (3 + 3) adenocarcinoma. The patient was directed to an active surveillance protocol, and (f) to (j) images show a follow-up MRI performed 2 years after the previous one. (f, j) Axial and coronal T2WI show the same lesion, which resulted in an increase in dimension (white arrow). (h, i) DWI (*b* = 2000) and the ADC map demonstrate a focal diffusion restriction, increased compared to the previous MRI (white arrow). (g) DCE imaging shows early and focal enhancement (white arrow). The lesion was classified as PRECISE 4. The lesion was re-biopsied, demonstrating the presence of clinically significant prostate cancer, ISUP 2 (GS 3 + 4) on 64% of the core length. The image quality was optimal for both the exam, and the examination was graded as PI-QUAL 3/3 according to PI-QUAL v2. Abbreviations: ADC = apparent diffusion coefficient map, DCE = dynamic contrast enhancement, DWI = diffusion-weighted imaging, PI-QUAL = prostate imaging quality, PI-RADS = prostate imaging-reporting and data system, T2WI = T2-weighted imaging.

#### Diagnostic accuracy and clinical impact

This system considers alterations in lesion morphology, size, conspicuity, diffusion characteristics, the emergence of new lesions, and the stage migration. PRECISE thus transforms MRI from a static diagnostic tool into a dynamic biomarker of temporal tumor behavior.^23^^,^^24^

Evidence supporting the clinical value of PRECISE score is substantial. Indeed, cases classified as PRECISE score 4-5 on confirmatory MRI within 18 months after diagnosis were associated with a 3-fold higher risk of csPCa progression after 4 years.^25^ Importantly, PRECISE does not replace confirmatory biopsy, which remains essential during early surveillance; rather, it helps refine which patients may safely defer rebiopsy.^26^ These findings have been reinforced in a multicenter validation of 1.667 patients on active surveillance, in which a PRECISE score of 4-5 conferred a 4.53-fold higher odds of biopsy progression to Grade Group ≥2 (95% CI, 3.37-6.12; *P* < 0.001); at a threshold of PRECISE ≥4 the system achieved a sensitivity of 57%, specificity of 79%, positive predictive value of 46%, and negative predictive value of 85%.^27^

#### Limitations and areas of development

Although PRECISE improves interpretability and reduces interobserver variation, challenges remain. The score relies on qualitative descriptors that lack quantitative thresholds, lesion measurement methods are not standardized, and MRI variability across centers can affect comparability between scans, even from the same patient; these limitations can lead to inconsistencies in interpretation.^26^ Reported reproducibility among dedicated readers is nonetheless substantial: a 2-center study described a per-patient Cohen κ of 0.71, rising to 0.83 when scores were grouped by the absence vs presence of radiological progression.^28^

Nonetheless, PRECISE represents a major step toward precision monitoring in AS, and ongoing work aims to integrate quantitative imaging biomarkers and artificial intelligence (AI) to improve reproducibility.

## Post-treatment phase

### PI-RR: assessing the risk of post-treatment local recurrence

#### Structure and principles

Following definitive treatment such as radical prostatectomy or radiation therapy, recurrence remains a significant clinical problem. Many patients experience rising PSA levels (biochemical recurrence) that necessitates imaging evaluation to determine whether the recurrence is local, regional, or distant. Although Prostate-Specific Membrane Antigen Positron Emission Tomography/Computed Tomography (PSMA PET/CT) has significantly improved accuracy for systemic recurrence, MRI remains essential for assessing local recurrence in the prostate bed or within the irradiated gland. However, post-treatment anatomy is complex, and conventional PI-RADS criteria are not appropriate for recurrence evaluation. The PI-RR (Prostate Imaging-Recurrence Reporting) scoring system was developed to address this gap by standardizing MRI interpretation after treatment.^29^

PI-RR score provides a structured 1-5 scale reflecting the likelihood of local recurrence. After radical prostatectomy, the prostate bed is evaluated primarily with DCE imaging, as recurrent tumor typically demonstrates early and focal enhancement. DWI may assist but is often limited by susceptibility artifacts from surgical clips (Figure 3). After radiation therapy, both DWI and DCE become essential, as irradiated tissue demonstrates heterogeneous appearances due to fibrosis, necrosis, and vascular remodeling (Figure 4). PI-RR offers specific morphological and functional criteria to differentiate benign post-treatment changes from recurrent malignancy.^29^

**Figure 3:**
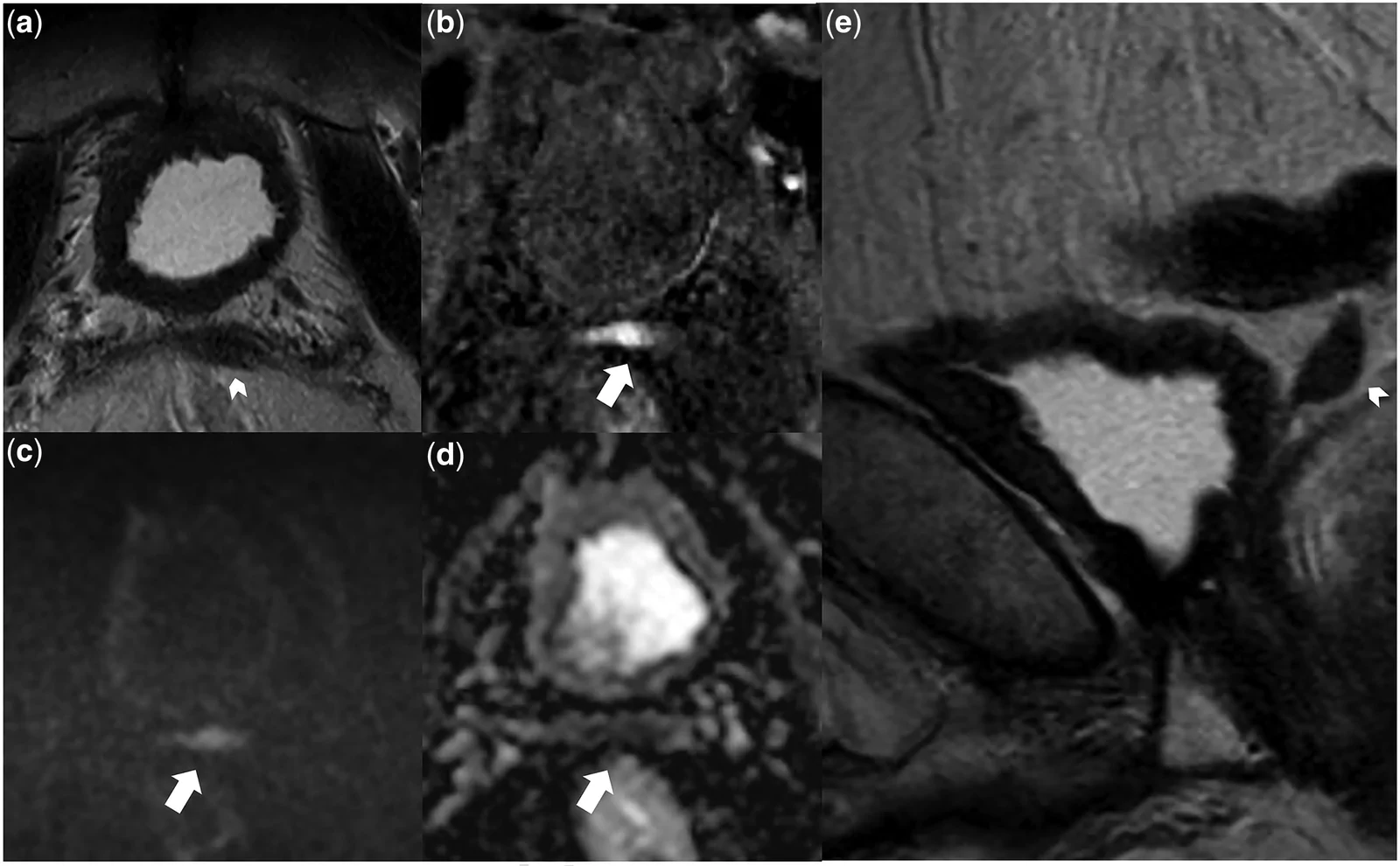
A 67-year-old patient with clinical suspicion of biochemical recurrence (PSA 1.11 ng/mL), who had undergone radical prostatectomy for prostate cancer Gleason score 7 (3 + 4) 3 years before the presented MR images acquisition. (a, e) Axial and sagittal T2-weighted images show solid tissue in the left seminal vesicle bed (white arrow). (c, d) DWI (*b* = 2000) and the ADC map demonstrate restricted diffusion (white arrow). (b) DCE imaging shows early and focal enhancement (white arrow). The abnormality was classified as PI-RR 5, as the lesion was located at the site of the primary tumor. Image quality according to PI-QUAL v2 was 3/3. Abbreviations: ADC map = apparent diffusion coefficient map, DCE = dynamic contrast enhancement, DWI = diffusion-weighted imaging, MRI = magnetic resonance imaging, PI-QUAL = prostate imaging quality, PI-RR = prostate imaging for recurrence reporting system.

**Figure 4:**
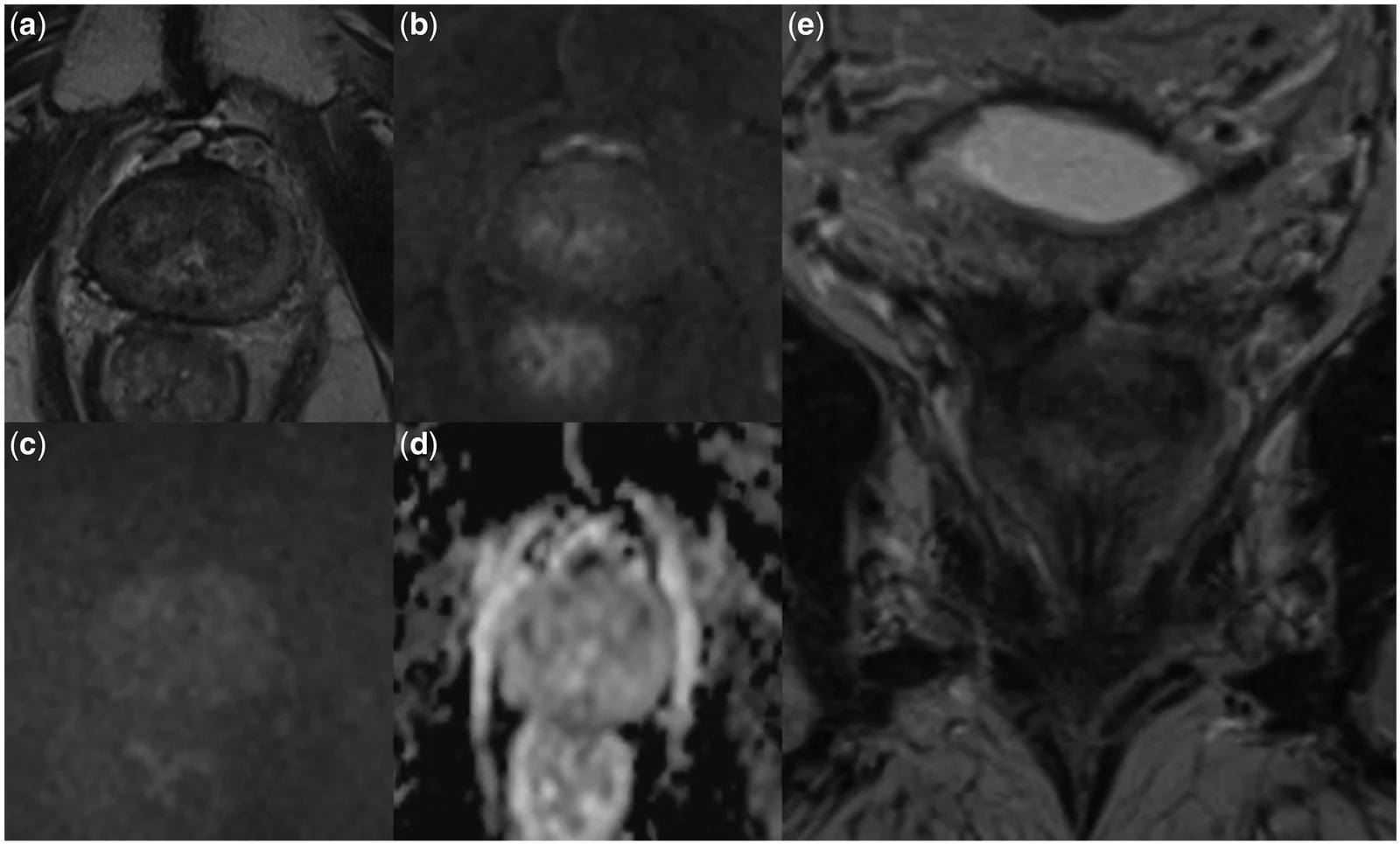
A 74-year-old patient who underwent radiation therapy and presented with clinical suspicion of recurrence (PSA 0.6 ng/mL); he underwent a follow-up MRI after 2 years. (a, e) Axial and coronal T2-weighted images show diffuse hypointensity of the peripheral zone due to post-radiotherapy changes, without any focal abnormality. (c, d) DWI (*b* = 2000) and the ADC map demonstrate no focal diffusion restriction. (b) DCE imaging shows homogeneous enhancement without focal areas of early uptake. The imaging findings were classified as PI-RR 1, indicating no evidence of recurrence. Image quality was optimal, and the examination was graded as PI-QUAL 3/3 according to PI-QUAL v2. Abbreviations: ADC map = apparent diffusion coefficient map, DCE = dynamic contrast enhancement, DWI = diffusion-weighted imaging, MRI = magnetic resonance imaging, PI-QUAL = prostate imaging quality, PI-RR = Prostate Imaging for Recurrence Reporting System.

#### Diagnostic accuracy, clinical impact, and limitations

Studies evaluating PI-RR score indicate that the system improves inter-reader consistency and correlates strongly with recurrence. Validation in multicenter cohorts shows that PI-RR scores of 4-5 are significantly associated with histologic confirmation of recurrence, whereas scores of 1-2 correlate with benign post-treatment normal changes.^30^ The introduction of PI-RR also supports more appropriate use of PSMA PET/CT. Indeed, patients classified by the European Association of Urology (EAU) recommendations as having a low likelihood of recurrence and a favorable prognosis can be appropriately assessed with MRI as the initial imaging modality, given their minimal risk of distant metastatic disease. On the other hand, individuals with intermediate or high risk of biochemical recurrence and less favorable prognostic features should ideally undergo PSMA-PET/CT as the preferred imaging approach.^31^ A subsequent systematic review and meta-analysis (6 studies, 467 patients) quantified this performance, reporting a pooled sensitivity of 77.8% (95% CI, 69.9-84.1%) and specificity of 80.2% (95% CI, 58.2-92.2%) for a PI-RR threshold of ≥3, and a sensitivity of 61.9% (95% CI 35.6-82.7%) and specificity of 86.6% (95% CI 75.1-93.3%) at a threshold of ≥4.^32^

However, wider validation for this promising tool is still needed to implement its wider spread.

### PI-FAB and TARGET: assessing risk of post focal therapy recurrence

#### Principles

Focal therapy (FT) represents an emerging management option for selecting men with localized prostate cancer, aiming to treat only the cancerous portion of the gland while preserving urinary and sexual function. Techniques include high-intensity focused ultrasound (HIFU), cryotherapy, irreversible electroporation (IRE), and photodynamic therapy. As the use of FT expands, imaging assumes a central role in patient selection, treatment planning, and post-treatment surveillance.^33^ However, MRI interpretation after focal therapy is complex. Treated zones exhibit variable appearances depending on the modality used, including necrosis, edema, fibrotic change, rim enhancement, or mass effect. Conventional PI-RADS criteria are not applicable to the treated gland.^33^

#### Prostate imaging-focal ablation

The PI-FAB (prostate imaging-focal ablation) scoring system was introduced to provide a structured evaluation of post-ablation MRI. PI-FAB categorizes imaging findings related to ablation zones, expected post-treatment effects, and features suspicious for residual or recurrent disease.^34^ Although the system is in early stages of development, it addresses a crucial and growing clinical need. PI-FAB uses a structured, 3-step approach to evaluate post-focal therapy MRI, beginning with the DCE sequences, followed by assessment of DWI (first the high-*b*-value images, then the ADC map) and concluding with T2WI. For accurate interpretation, access to the baseline pretreatment MRI is crucial. A PI-FAB score of 1 generally supports the continuation of standard monitoring without immediate intervention. When the PI-FAB score is 2, closer evaluation of PSA kinetics becomes important; a biopsy may be appropriate if PSA levels are rising, whereas stable biochemical findings typically justify postponing further investigations and scheduling the next MRI after about a year. In contrast, a PI-FAB score of 3 indicates a higher level of suspicion and should lead to a recommendation for biopsy.^34^

Initial validation studies show promising interobserver agreement, especially in identifying radiologic features of incomplete ablation or residual enhancing nodules suggestive of persistent cancer.^35^ Recently, a dedicated PSMA Focal Ablation Imaging (PSMA-FAB) scoring system has been proposed.^36^

#### TARGET

The Transatlantic Recommendations for Prostate Gland Evaluation with MRI after Focal Therapy (TARGET) represent another international consensus-based scoring system developed to standardize prostate MRI acquisition, interpretation, and reporting following FT. By providing dedicated guidance tailored to the post-FT prostate, TARGET aims to improve the detection of residual or recurrent clinically significant prostate cancer while reducing unnecessary biopsies and inter-reader variability. TARGET recommends the use of high-quality multiparametric MRI compliant with PI-RADS v2.1 technical standards, mandating the inclusion of T2WI, DWI, and DCE imaging.^13^ Surveillance MRI is advised at 12 months after focal therapy, with subsequent annual imaging based on MRI findings, PSA kinetics, and clinical risk factors. Interpretation should always be performed in comparison with the most recent pretreatment MRI, and structured reporting is encouraged to ensure consistency and clinical relevance.^37^ Lesions identified within the ablation zone are assessed using a lesion-based 5-point TARGET score that reflects the likelihood of recurrent disease. The scoring system follows a hierarchical algorithm in which DCE imaging is designated as the dominant sequence. Each sequence is individually scored on a 3-point scale (1 = nonsuspicious, 2 = equivocal, 3 = suspicious), and these scores are combined to derive an overall TARGET score ranging from 1 (very low suspicion) to 5 (very high suspicion). Scores of 4 or 5 are considered highly suggestive of recurrent cancer and generally warrant targeted biopsy, whereas scores of 1-2 support routine surveillance and score 3 indicates indeterminate findings requiring closer follow-up or additional imaging.^37^^,^^38^ Clinical validation of TARGET remains limited, but preliminary evidence suggests that it performs comparably to other post-focal therapy MRI scoring systems. In the first head-to-head comparison, TARGET and PI-FAB demonstrated similar overall diagnostic accuracy for detecting recurrent clinically significant prostate cancer after focal therapy.^39^

## Staging phase

### Extraprostatic extension and local (T) staging

Radiological T-staging with the detection of extraprostatic extension (EPE) remain among the least standardized areas of prostate MRI, which distinguishes organ-confined (T2) from locally advanced (T3a) disease imperfectly and with marked interobserver variability.^40^ Several scoring systems have been proposed, none achieving consensus or clear prognostic superiority^40^ such as: the ESUR EPE score, the first structured 5-tier framework based on qualitative signs^41^; the NCI mEPE-grade, which added quantitative curvilinear capsular contact length to a 3-tier scheme^42^; the Early-and-late-EPE score, arranging 8 T2-weighted criteria along a biological continuum^43^; the 5-tier mEPE-score^44^; and the simplified sEPE grading, based on capsular contact length (≥15 mm) and bulging/irregularity.^45^ Across systems, tumor volume and capsular contact length are the most reproducible and specific predictors, whereas most features show limited sensitivity and only moderate agreement (areas under the curve, AUCs, ∼0.70-0.80).^40^

### Node reporting and data system

Node reporting and data system (Node-RADS) is a standardized classification system designed to evaluate lymph node involvement in oncologic imaging.^46^ By combining traditional size measurements with detailed morphological and structural features, such as internal texture, contour irregularity, and nodal shape, it provides a more comprehensive assessment of suspicious lymph nodes. Scores range from 1 to 5, indicating increasing levels of malignancy risk and promoting greater consistency and accuracy in MRI or CT reporting. Current evidence in prostate cancer suggests that Node-RADS is particularly useful in confirming nodal disease; however, its limited sensitivity reduces its effectiveness in detecting all metastatic nodes, preventing it from replacing established clinical prediction tools for local staging in prostate cancer.^47^ In a recent multicenter study investigating a cohort of patients with prostate cancer and treated with radical prostatectomy and extended pelvic lymph node dissection, Node-RADS proved high specificity (99.3%) and positive predictive value (50.6%), a key tool to confirm nodal involvement. Moreover, Node-RADS overperformed PSMA-PET/CT in overall accuracy, with a 0.637 vs 0.526 AUCs; on the other hand, combining Node-RADS with the already validated Briganti 2019 nomogram achieved the strongest performance (AUC 0.719), compared to PSMA-PET/CT and to the nomogram alone.^48^^,^^49^

### Metastasis reporting and data system for prostate cancer

The Metastasis reporting and data system for prostate cancer (MET-RADS-P) provides a standardized approach for the acquisition, interpretation, and reporting of whole-body magnetic resonance imaging (WB-MRI) in patients with advanced prostate cancer.^50^ Designed to reduce technical and analytical variability across institutions, MET-RADS-P establishes minimum imaging parameters, promotes a multi-sequence approach to lesion assessment, and introduces structured reporting templates that classify metastatic involvement across bone, nodal, visceral, and local disease sites. Central to the system is a 5-point regional response scale that captures the degree of therapeutic response or progression, including the ability to document heterogeneous or discordant patterns among metastatic sites. By enabling consistent classification of disease burden, response dynamics, and the temporal evolution of metastases, MET-RADS-P supports both clinical decision-making and the integration of WB-MRI into therapeutic trials, addressing the need for objective and reproducible imaging biomarkers in advanced prostate cancer management. In MET-RADS-P, the assessment of response categories is performed at a regional rather than a purely lesion-based level, reflecting the biological heterogeneity typically seen in advanced disease. Following WB-MRI acquisition, lesions are reviewed across multiple sequences (T1-weighted, fat-suppressed T2/STIR, Dixon reconstructions, high *b*-value DWI, and ADC maps), and grouped into anatomical regions encompassing the primary site, 7 skeletal segments, 5 nodal and visceral compartments, and an additional “other” category. Each region is evaluated using quantitative and qualitative features, such as changes in lesion size, high *b*-value signal intensity, ADC shifts consistent with viable tumor or necrosis, marrow reconversion, or fat deposition within lesions. Based on these features, each region is assigned a Response Assessment Category (RAC) on a 5-point scale: from highly likely responding to highly likely progressing. The dominant pattern of change within each region defines the primary RAC, while secondary and, if present, tertiary patterns are recorded to capture intra-regional discordance without inflating progression calls based on isolated lesions. In doing so, MET-RADS-P integrates lesion heterogeneity into structured response assessment, ensuring a balanced representation of both treatment efficacy and biologically relevant resistance processes. In the only dedicated inter-observer study to date, agreement between readers of differing expertise for the primary regional response category ranged from fair at the primary site (κ  =  0.21) to excellent throughout the skeleton (*k* from 0.78 to 0.93), whereas agreement on the resulting patient-level management decision was almost perfect (Cohen κ  =  0.92; 96.8% concordance).^51^ Moreover, a recent comparison in 184 men with metastatic castration-resistant prostate cancer found only moderate concordance between MET-RADS-P and the reference Prostate Cancer Clinical Trials Working Group 3 (PCWG3) criteria (weighted κ  =  0.48, and just κ  =  0.40 in bone), with MET-RADS-P detecting progression earlier and earlier progression predicting worse survival; indeed, early MET-RADS-P progression was significantly associated with worse overall survival (adjusted hazard ratio = 4.75; 95% CI = 2.1-10.7; *P* < .001).^52^

### Molecular imaging (PSMA-RADS)

The advent of prostate-specific membrane antigen (PSMA) radiotracers has revolutionized molecular imaging in prostate cancer. PSMA PET/CT and PET/MRI exhibit strong sensitivity for detecting metastatic disease, recurrent lesions at low PSA levels, and nodal involvement. These advances have rapidly reshaped staging algorithms and recurrence work-up. However, as with MRI, there is a risk of interpretive variability without a standardized lexicon.^53^

The Prostate-Specific Membrane Antigen—Reporting and Data System (PSMA-RADS) was developed to address this need. PSMA-RADS provides a structured 1-5 scoring system reflecting the likelihood that a PSMA-avid lesion represents prostate cancer metastasis or recurrence.^54^ A notable feature of PSMA-RADS is its inclusion of subcategories for equivocal findings, enabling clinicians to differentiate between benign PSMA uptake and indeterminate lesions requiring further evaluation.

Robust evidence demonstrates an excellent correlation between PSMA-RADS categories and both histological confirmation and clinical outcomes. Large studies highlight the high diagnostic accuracy of PSMA PET, particularly for nodal metastases and oligometastatic recurrence.^55^ PSMA-RADS therefore serves as a critical tool for standardizing reporting across institutions and guiding decisions regarding metastasis-directed therapy, systemic treatment escalation, or salvage interventions. Quantitatively, the prospective trial underlying these categories reported a positive predictive value of 0.84 for PSMA-PET on a per-patient basis against histopathology, and 0.92 when validated against a composite reference standard.^55^

As hybrid PET/MRI platforms become more widely used, PSMA-RADS may integrate more tightly with MRI-based scoring systems, creating multimodal frameworks.

Beyond PSMA-RADS, several complementary approaches now structure PSMA-PET interpretation.^56^ For primary staging, the PRIMARY score grades intraprostatic disease on a 5-point scale based on the pattern and intensity of PSMA uptake,^57^ whereas the molecular imaging TNM (miTNM) classification, consolidated within the second version of the Prostate Cancer Molecular Imaging Standardized Evaluation framework (PROMISE V2), standardizes whole-body staging, PSMA-expression scoring, and eligibility assessment for radioligand therapy.^58^ For treatment monitoring, the Response Evaluation Criteria in PSMA-PET/CT (RECIP 1.0) classify response by integrating the emergence of new lesions with the change in total PSMA-positive tumor volume,^59^ while the PSMA-PET Progression (PPP) criteria define progression primarily through the appearance of new lesions.^60^

## PI-QUAL: assessing prostate MRI quality

### Structure and principles

As multiparametric MRI became central to prostate cancer diagnosis, an important limitation became increasingly apparent: the diagnostic reliability of any imaging-based scoring system is inherently constrained by the quality of the underlying images. Even the most experienced radiologist cannot accurately interpret sequences degraded by motion, susceptibility artifacts, insufficient resolution, or inadequate signal-to-noise ratios (SNR).^61^ Such variability is not rare; MRI quality differs widely across institutions, scanners, operators, and patient factors.^62^ These disparities complicate both research and clinical care, and may undermine the performance of PI-RADS, particularly in lower-volume centers.^63^ To address these challenges, the Prostate Imaging Quality (PI-QUAL) v1 scoring system was developed as a structured method for assessing whether a prostate MRI examination meets the quality standards required for meaningful interpretation.^9^ A recent update (PI-QUAL v2) of the scoring system has been published,^64^ and replaces the earlier 5-point scale with a 3-level system that grades prostate MRI according to the quality of T2WI and DWI and, when used, the adequacy of DCE sequences. A scan is automatically inadequate (score 1) when essential criteria are not met, or when poor SNR, inability to delineate key prostate structures, significant motion or susceptibility artifacts, or mismatching/distorted diffusion images affect the images. A score of 2 reflects acceptable quality in which T2WI and DWI meet some of these criteria (adequate SNR, clear anatomical definition, clear ADC maps, and minimal artifact). A score of 3 indicates optimal image quality: sharp and well-delineated T2WI and distortion-free high-b DWI with high-quality ADC maps (Figure 5). These focused criteria help identify the specific technical limitations that degrade image quality and enable corrective action during acquisition, improving diagnostic consistency across both contrast and non-contrast prostate MRI. Indeed, when image quality is optimal, this enables high-confidence rule-in and rule-out decision-making.^64^

**Figure 5:**
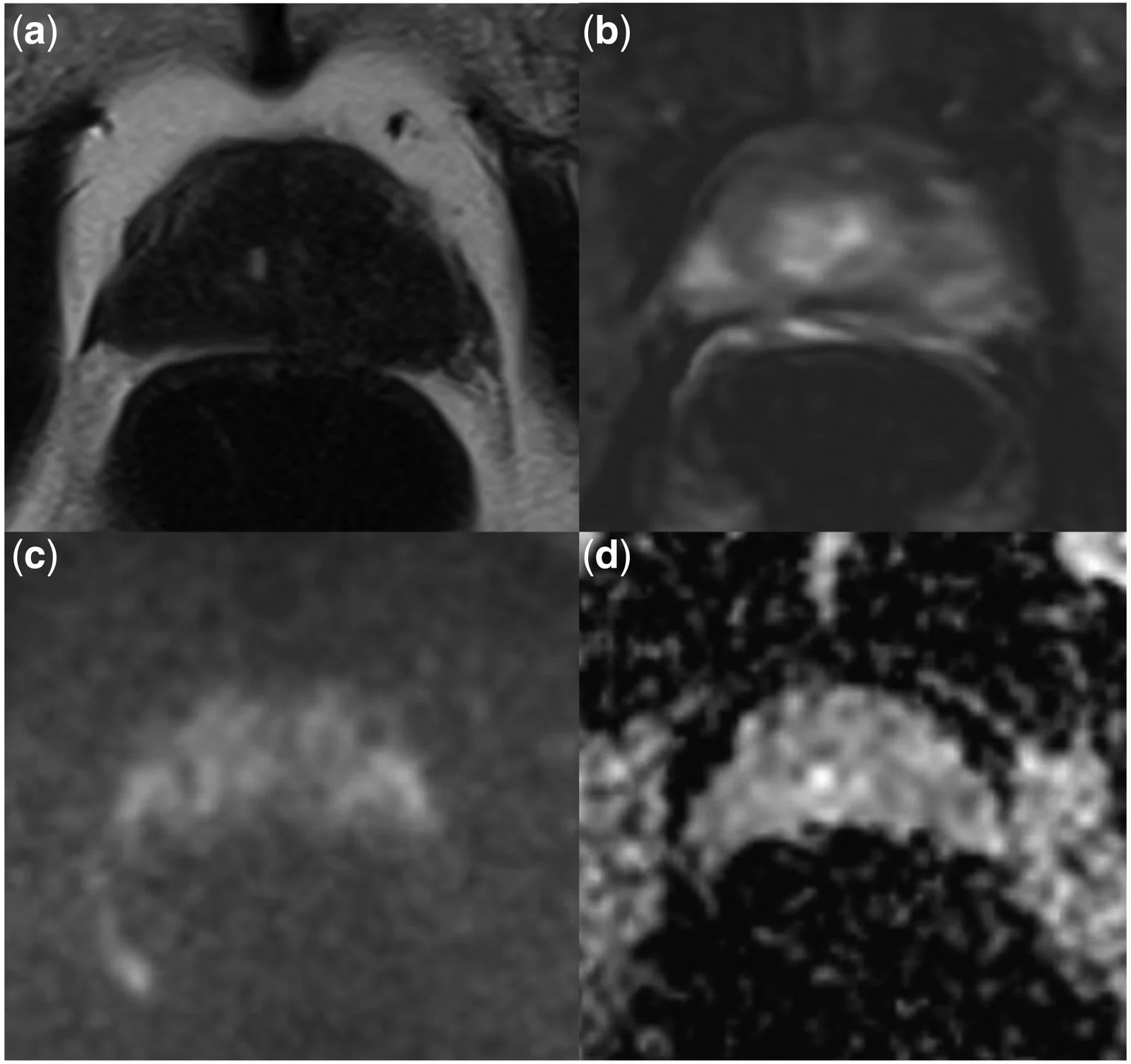
This multiparametric MRI demonstrates artifacts caused by the presence of air in the rectum, visible on DWI and ADC map; the examination was classified with a PI-QUAL 2/3. Abbreviations: ADC = apparent diffusion coefficient map, DWI = diffusion-weighted imaging, MRI = magnetic resonance imaging, PI-QUAL = prostate imaging quality.

### Clinical impact and limitations

The development of PI-QUAL also reflects broader trends toward standardization in oncologic imaging, where image quality proved to directly influence diagnostic performance, eg, considering lung and breast cancer screening.^65^^,^^66^ In prostate cancer care, PI-QUAL is likely to become an indispensable complement to PI-RADS, particularly as MRI becomes widely used in screening and early detection initiatives.^67^

Emerging data using PI-QUAL v2 confirm that image quality translates directly into diagnostic performance: in a prospective study of 246 patients, optimal-quality examinations (PI-QUAL 3) achieved significantly higher accuracy for csPCa than lower-quality scans (AUC 0.79 vs 0.66; *P* = .01)—an approximately 15% gain—while the proportion of indeterminate PI-RADS 3 findings fell progressively from 27% at PI-QUAL 1% to 15% at PI-QUAL 2 and 8% at PI-QUAL 3.^68^

Future iterations may incorporate quantitative metrics, automated quality-check algorithms, and AI assistance to minimize subjective variability.

## Barriers and future perspectives

Despite the substantial progress achieved through structured prostate imaging interpretation systems, several important challenges remain for the optimization and standardization of prostate cancer imaging. Prostate MRI continues to evolve rapidly, influenced by advances in hardware, molecular imaging, and computational science. The scoring systems previously described represent essential steps toward standardization (Table 1). However, each system also highlights the inherent complexity of prostate imaging and underscores the need for continued refinement.

**Table 1:** Summary of the main scoring system applied to prostate imaging.

Scoring system	Primary purpose	Clinical context	Key features	Strengths	Limitations	Score	Score description
**Diagnostic and monitoring phase**							
**PI-RADS v2.1**	Risk assessment of clinically significant PCa	Men with clinical suspicion of PCa	T2WI, DWI/ADC, optional DCE; sector-based scoring	Strong evidence, widely validated, standardizes MRI interpretation; guides biopsy	PI-RADS 3 ambiguity; interobserver variability (especially transitional zone lesions); semi-subjective assessment; limited integration with clinical parameters	1-5	1-2: low suspicion; 3: equivocal; 4-5: high suspicion
**PRECISEv2**	Monitoring progression in active surveillance	Serial MRI during active surveillance	Changes in lesion size, conspicuity, DWI/ADC and DCE behavior	Structured follow-up; avoids unnecessary biopsies; widely adopted in active surveillance trials	Lack of strict quantitative thresholds; subjective progression assessment; limited integration with clinical variables; limited large-scale validation	1-5	1-2: regression; 3: unchanged; 4-5: progression
**Post-treatment phase**							
**PI-RR**	Detection of recurrence after local treatment	Post-treatment (radical prostatectomy, radiation therapy)	DCE, DWI, location-based patterns	Supports salvage therapy decisions; structured reporting	Complex anatomy post-treatment; limited validation; variability in DCE interpretations	1-5	1-2, benign post-treatment changes; 3, indeterminate; 4-5, increasing likelihood of local recurrence
**PI-FAB**	Assessment of suspected recurrence after focal therapy	Post-focal therapy MRI	DWI, DCE; patterns of focal ablation zones	Addresses major gap in post-focal therapy assessment	Requires further validation; limited reproducibility data; learning curve; vendor-specific DCE issues	1-5	1: fibrosis; 2: vessel/inflammation; 3: suspicious; 4-5: recurrent disease
**TARGET**	Assessment of suspected recurrence after focal therapy	Post-focal therapy MRI	Multi-sequence MRI; emphasis on DCE	Standardized recurrence scoring; fills gap for post-treatment imaging	Early stage of dissemination; lacks large multi-institution validation; potential subjectivity in DCE	1-5	1-2: benign; 3: indeterminate; 4-5: high suspicion
**Staging phase**							
**Node-RADS**	Structured nodal evaluation on CT/MRI	Nodal staging	Size, shape, border characteristics	Standardizes nodal assessment; improves clarity in reports	Low sensitivity for micro-metastases; no functional imaging; limited validation	1-5	Low → high risk of nodal metastasis
**MET-RADS-P**	WB-MRI response assessment	Advanced/metastatic PCa; clinical trials	Multi-sequence WB-MRI; lesion response patterns	Captures spatial heterogeneity; strong for clinical trial endpoints	Time-consuming; requires expertise; limited accessibility; interobserver variability; not widely validated across centers	1-5	Likelihood and degree of metastatic progression
**PSMA-RADS**	Interpretation of PSMA PET-CT	Primary staging and biochemical recurrence	PSMA uptake pattern, anatomic correlation	High sensitivity for metastases; structured PET interpretation	Benign uptake pitfalls; limited integration with MRI/clinical parameters; interobserver variability; not equally accessible in low-resource settings	1-5	Spectrum from benign to malignant uptake
**Quality assurance**							
**PI-QUAL v2**	Quality assessment of prostate MRI	All prostate MRI exams	Protocol adherence, artifacts, coverage, resolution	Improves interpretability, supports quality assurance programs	Not widely adopted; interobserver variability; requires training; less feasible in low-resource settings	1-3	1 poor quality; 2 adequate; 3 optimal

Abbreviations: ADC = apparent diffusion coefficient, DCE = dynamic contrast enhancement, DWI = diffusion weighted imaging, MET-RADS-P = Metastasis Reporting and Data System for Prostate Cancer, Node-RADS = node reporting and data system, PCa = prostate cancer, PI-FAB = prostate imaging-focal ablation, PI-RADS = prostate imaging reporting and data system, PI-RR = prostate imaging for local recurrence reporting, PRECISE = prostate cancer radiological estimation of change in sequential evaluation, PSMA = prostate-specific membrane antigen, PSMA-RADS = prostate-specific membrane antigen—reporting and data system, T2WI = T2-weighted imaging, TARGET = transatlantic recommendations for prostate gland evaluation with mri after focal therapy.

This challenge is particularly evident in MRI-based T-staging. Over the past decade, multiple extraprostatic extension scoring frameworks have been proposed, but their adoption has been inconsistent across institutions. Current tools differ in scale, rely heavily on subjective visual criteria, and lack robust multicenter validation, leading to heterogeneity in how radiologists assess extraprostatic extension with therapeutic implications for the patient. With the rapid evolution of MRI technologies and modern treatment techniques, there is growing recognition that T-staging scoring systems must be updated and clinically aligned.^40^

One of the most pressing barriers is inter-reader variability, which persists across all prostate imaging scoring systems despite training initiatives and consensus guidelines.^28^^,^^32^^,^^35^^,^^51^^,^^69^^,^^70^ While expert radiologists demonstrate high concordance, performance in general practice is less consistent, particularly for transition-zone lesions, PI-RADS 3 cases, and post-treatment imaging. This variability reduces the reliability of MRI-based pathways and may contribute to differences in biopsy decision-making and treatment recommendations between institutions. Addressing this challenge requires not only further standardization but also expansion of structured education programs, certification pathways, and proficiency assessments.

A second barrier relates to MRI accessibility and resource variability, which affect the implementation of structured scoring systems worldwide. High-quality mpMRI requires advanced scanners, optimized protocols, and experienced technologists, all of which may be limited in low-resource settings. MRI without contrast medium has been proposed as a solution, offering reduced cost, shorter scan times, and no requirement for contrast administration.^21^ Addressing global disparities in imaging resources will be key to ensuring equitable access to high-quality prostate cancer diagnostics.

A third challenge lies in the setting of post-treatment imaging, where an increasing array of therapeutic options complicates interpretation. The morphology of the prostate after radiotherapy, ablation, or surgery varies dramatically.

Another evolving frontier is hybrid and multimodal imaging, particularly PSMA PET/MRI. PSMA-PET/CT has demonstrated promising diagnostic accuracy for recurrence and has already influenced staging algorithms in intermediate- and high-risk disease. The integration of PET with MRI offers synergistic benefits by combining molecular specificity with high-resolution anatomic and functional imaging. However, the coexistence of MRI-based and PET-based scoring systems poses interpretive challenges. Over time, there is likely to be convergence toward more integrated scoring systems, capable of synthesizing morphological, functional, and molecular biomarkers into a unified risk stratification scheme.^71^

The incorporation of AI represents one of the most transformative future directions.^72^ AI has already shown promise in automated prostate segmentation, lesion detection, PI-RADS categorization, quality assurance assessment, and prediction of clinically significant cancer.^72–75^ Machine-learning models using radiomic features extracted from mpMRI can distinguish aggressive from indolent tumors and may predict genomic risk scores or biochemical recurrence. Deep learning has been applied to denoising, motion correction, and artifact reduction, directly improving the quality of images before human interpretation.^76–78^ The strongest evidence to date comes from the PI-CAI study, a large international, paired, non-inferiority investigation in which a state-of-the-art AI system achieved an area under the receiver-operating-characteristic curve of 0.91 (95% CI, 0.87-0.94) vs 0.86 (95% CI, 0.83-0.89) for a panel of radiologists reading with PI-RADS.^72^ These results are compelling but warrant critical interpretation: they were obtained in a retrospective, reader-study setting on curated multicenter data, so prospective clinical validation, demonstration of generalizability across scanners and populations, and vigilance regarding automation bias remain crucial prerequisites. Importantly, AI offers the potential to democratize access to expert-level MRI performance by providing decision support to less-experienced readers, thereby reducing variability and improving diagnostic confidence.^69^^,^^79^^,^^80^

Another key frontier is the integration of imaging biomarkers with clinical and genomic data. Prostate cancer is a heterogeneous disease, and imaging increasingly reflects its molecular underpinnings. Studies have demonstrated correlations between MRI features, such as ADC values, lesion conspicuity, and perfusion kinetics, and tumor genomics, such as micro-RNA. Combining imaging-derived markers with molecular classifiers could yield hybrid prediction models with greater precision than either data source alone.^81^^,^^82^

A final barrier concerns the need for prospective multicenter validation of emerging scoring systems, to demonstrate generalizability across populations, scanners, and treatment modalities.

Looking ahead, the future of prostate imaging scoring systems will depend on 3 overarching directions: (1) harmonization, through unified approaches that reduce variability and reconcile differences across scoring systems (Figure 6); (2) digital augmentation, integrating AI and hybrid imaging into coherent multimodal frameworks; and (3) clinical personalization, tailoring imaging pathways to individual patient risk, disease biology, and treatment goals.

**Figure 6:**
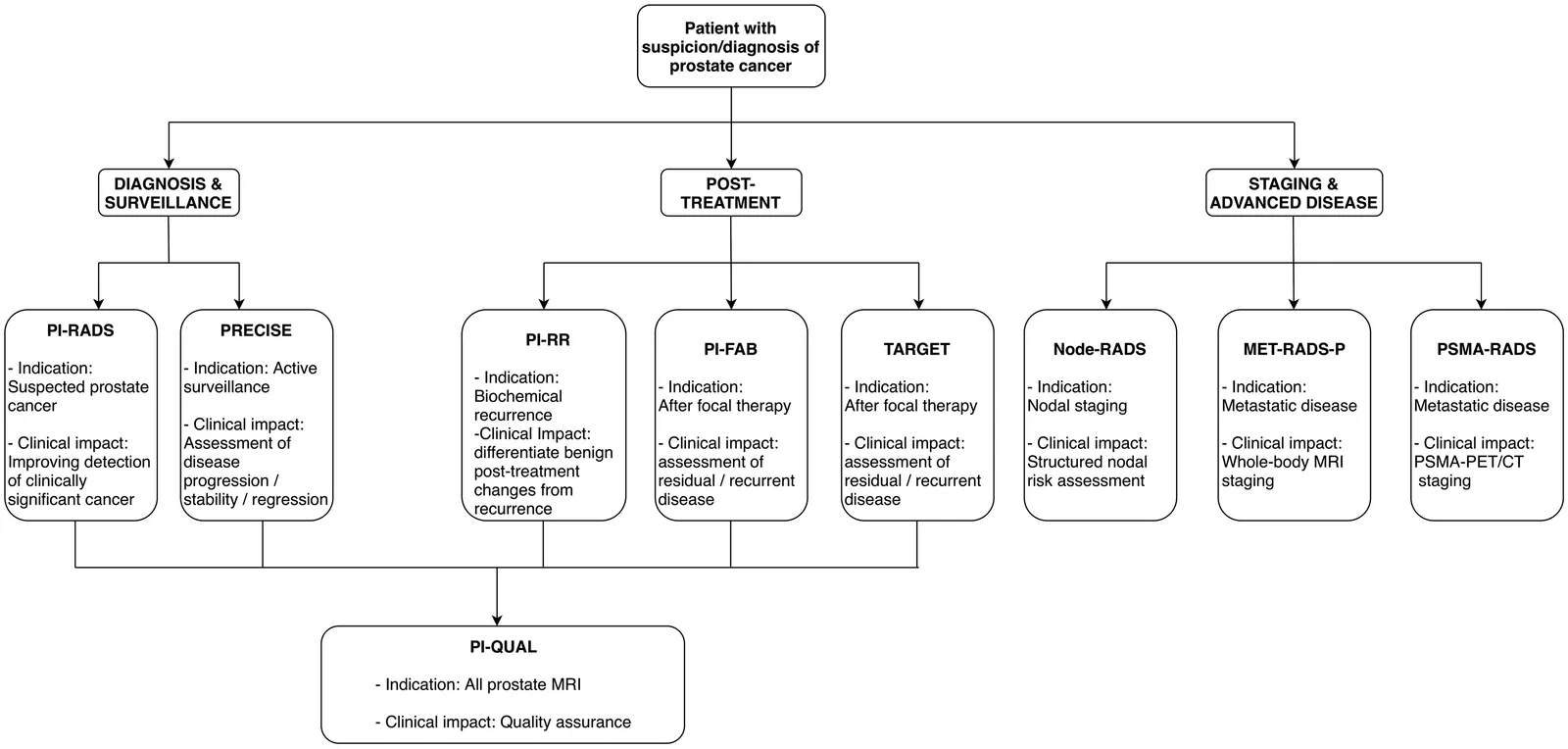
Schematic flowchart of the different scoring systems applied to prostate imaging according to specific clinical indications. Abbreviations: MET-RADS-P = metastasis reporting and data system for prostate cancer, MRI = magnetic resonance imaging, Node-RADS = node reporting and data system, PI-FAB = prostate imaging-focal ablation, PI-RADS = prostate imaging reporting and data system, PI-RR = prostate imaging for local recurrence reporting, PRECISE = prostate cancer radiological estimation of change in sequential evaluation, PSMA PET/CT = prostate-specific membrane antigen positron emission tomography/computed tomography, PSMA-RADS = prostate-specific membrane antigen—reporting and data system, TARGET = transatlantic recommendations for prostate gland evaluation with MRI after focal therapy.

## Conclusions

The development of structured reporting systems of prostate advanced imaging modalities has fundamentally reshaped the diagnostic and therapeutic landscape of prostate cancer. The reporting systems have introduced a consistent lexicon, improved inter-reader agreement, and provided clinicians with reproducible criteria to support decision-making across the entire patient pathway, from early detection to treatment response assessment and metastatic disease evaluation. The ongoing evolution of imaging-based scoring systems will enable more precise, tailored, and biologically informed management of prostate cancer, strengthening their role as indispensable components of modern oncologic practice.
